# Light Microscopy as a Tool to Evaluate the Functionality of Starch in Food

**DOI:** 10.3390/foods9050670

**Published:** 2020-05-22

**Authors:** Wioletta Błaszczak, Grażyna Lewandowicz

**Affiliations:** 1 Institute of Animal Reproduction and Food Research, Polish Academy of Sciences in Olsztyn, 10 Tuwima St., 10-748 Olsztyn, Poland; w.blaszczak@pan.olsztyn.pl; 2 Department of Biotechnology and Food Microbiology, Poznań University of Life Sciences, 48 Wojska Polskiego St., 60-627 Poznań, Poland

**Keywords:** starch, microscopy, structure, gelatinisation, modification

## Abstract

Light microscopy (LM) is commonly used in the study of biological materials to determine the morphology of cells and tissues. The potential of this technique for studying the structure of food products is also recognized but less known. Especially rare are information regarding LM studies of the supramolecular structure of starch. The aim of the work was to fill this gap by providing data on the possibilities for application of LM in starch studies. It was shown that in spite of an enormous progress in the development of microscopic techniques, including both increase of resolution and improvement of image analysis methods, light microscopy still has a huge potential for starch studies. The advantage of LM over other microscopic techniques is the possibility of differentiating between amylose and amylopectin by iodine staining. That makes LM especially useful in the analysis of the process of gelatinization of starch, the extent of molecular dispersion of its macromolecules, and the changes in its structure caused by modification. Moreover, it can be particularly useful for studying the changes in the supramolecular structure of starch in a food product matrix, providing more information than scanning electron microscopy (SEM)–the most common technique used for these purposes.

## 1. Introduction

The history of the use of microscopy in starch research dates back to the early days of microscopy in XVII century and is closely related to the findings of Antonie van Leeuwenhoek who confirmed the presence of starch in various parts of plants [1]. Moreover, his observations enabled preliminary estimation and comparison of shapes and sizes of starch granules of various species of plants [2]. However, this technique became common only in the 20th century. Optical microscopes of that time made it possible to obtain magnification factors of several hundreds and, in consequence, allowed the observation not only of the shape of starch granules but also of other certain morphological features such as the layered nature of internal structure of the granules (growth rings), hilum and equatorial groove. These structural elements were particularly possible to observe during swelling of the largest granules of wheat starch [3]. Further possibilities for microscopic starch research were opened by the introduction of scanning electron microscopy. Magnification of the order of thousands of times enabled the study of sub-granular structures and changes in the morphology of starch granules throughout their development or resulting from processing [4,5]. It was shown that fibrillar structures are formed by linearization. Thanks to the use of scanning electron microscopy, characteristic degradation patterns of granules of cereal starches resulting from hydrolysis catalysed by glucoamylase or α-amylase were established. Moreover, lower susceptibility of legume starches to enzymatic hydrolysis was found [6]. Further development of this area of research enabled the establishment of a more detailed supramolecular structure. So called ‘blocklet’ model of granules organisation has been proposed. According to it, the crystalline and amorphous lamellae of the amylopectin are organized into larger, more or less spherical structures termed ‘blocklets’. The blocklets range in diameter of about 20–500 nm depending on location in the granule and botanical source of starch. They play a significant role in the resistance of starch to enzymatic attack [7]. Another imaging technique that has been reported useful for structural analysis of starch is confocal laser scanning microscopy (CLSM). The structural elements can be visualised with different fluorophores. For example, rhodamine and safranin enabled detailed investigations of the growth ring structure of granules [8]. As indicated, CLSM may provide even more comprehensive insight into the internal granular structures than scanning electron microscopy (SEM). While both these imaging techniques are highly complementary, SEM remains the most convenient tool for studying the morphology of starch, including its surface and the granule interior [9]. A technique that utilizes electron beam imaging, transmission electron microscopy (TEM) provides information regarding internal architectural organization. TEM is a powerful technique that allows imaging of the biopolymer structures distinctly at higher resolution when compared to SEM or CLSM. On the other hand, one-dimensional imaging, typical for TEM, can narrow down to some extent the information on the internal structure of starch granules. Therefore, TEM has not found such wide application in the structural studies of starch.

The topography of granules i.e., presence of pores, channels or non-starch compounds, may distinctly modify the physicochemical properties of starch with emphasis on its susceptibility to chemical or physical treatment. Atomic force microscopy (AFM) has been successfully used to probe surface structures, determine the surface properties of native and/or modified starches (adhesion, elasticity) and the interactions of polymers at the nano-structural level [10,11,12]. AFM has also been applied to the study of the nanostructure of starch molecules and its changes upon gelatinization [13,14] or to the analysis of the formation of molecular networks by retrograded starch [15]. The starch structure, with emphasis on the crystal structure, can also be analysed using scanning acoustic microscopy (SPAM). The sonic wave employed to probe crystal structure at nano-scale makes this technology possible to be effectively employed alongside atomic force microscopy (AFM) [16].

The combination of various microscopic and/or spectroscopic techniques allows scientists to analyse not only the structural alterations but also the changes in the chemical profile of the processed granules [17,18]. On the other hand, studies on the microstructural changes induced by hydrothermal treatment, such as granule swelling and leakage of amylose, or studies on the relation of the rheological behaviour of starch systems and their microstructure were carried out with the use of light microscopy (LM) [19]. The advantage of LM lies in the possibility of differentiating between amylose and amylopectin by iodine staining. As a result, it is possible to study the distribution of amylose and amylopectin both in the dispersed and continuous phases of starch pastes [19]. However, huge progress in the development of microscopic techniques, including both the increase of resolution and improvement of image analysis methods, has left the oldest technique–light microscopy–somewhere on the sidelines of mainstream science. Especially rare in literature are information regarding LM studies of the supramolecular structure of starch and its changes resulting from modification.

The aim of this work was to fill this gap by providing data that regards the possibilities for application of LM in starch studies. The gelatinization process of the most popular commercial starches that differ in amylose content and in pasting viscosity profile was investigated. This made it possible to show that thickening capability is related to the extent of molecular dispersion of starch. Moreover, microstructure of the most important modified potato starches was studied (oxidised, acetylated, cross-linked and cationic). It was shown that the incorporation of hydrophilic and hydrophobic moieties into starch macromolecules affected not only the viscosity but also significantly changed the ability to complex iodine within amylose and amylopectin helixes. The application of light microscopy to the studies of cross-linked starch allowed to observe the effect of acids or the sterilisation process on the molecular dispersion of starch and the resulting applicability of different modified starches as texture forming agents for different food products.

## 2. Materials and Methods

### 2.1. Materials

Commercial native potato starch (ZETPEZET, Piła, Poland) was the initially studied material. Commercial pregelatinised drum dried potato starch (ZETPEZET, Piła, Poland), oxidised potato starches of different degree of substitution (DS) (WPPZ, Luboń, Poland) and native starches of other botanical origin i.e., tapioca super high grade (Thailand), wheat (Roquette), corn (Cargill) and waxy corn (Cargill) were also used in this study.

All modification processes were performed using potato starch as a raw material. Starch extrusion was performed in an industrial scale twin screw extruder (custom made) operating at temperature of 135 °C and 80 rpm. Initial moisture content of starch subjected to modification was 13%. The extrudate was grinded and sieved through a 60 mesh.

Acetylated starches were prepared in water suspension with acetic anhydride according ≥98% (Sigma-Aldrich Co., St. Louis, MO, USA) to the procedure described in [20]. Crosslinked starches were obtained in suspension using trisodium trimetaphosphate ≥95% (STMP, Sigma-Aldrich Co., St. Louis, MO, USA) as the crosslinking agent according to the procedure described in [21]. Cationic starches of DS = 0.04 were obtained in water suspension with 3-chloro-2-hydroxy-N,N,N-trimethylpropylammonium chloride 99% (Sigma-Aldrich Co., St. Louis, MO, USA) as cationising agent. Derivatives of DS = 0.13 and DS = 0.27 were obtained using dry microwave-assisted process with N,N,N-trimethyl-2-epoxypropylammonium chloride ≥90% (Sigma-Aldrich Co., St. Louis, MO, USA). Both procedures were described in [22].

### 2.2. Methods

Pasting characteristics were analysed with a Brabender viscograph under the following conditions: measuring cartridge 0.07 Nm; heating/cooling rate 1.5 °C/min; thermostating 20 min. pH value of starch suspensions subjected to viscographic analyses were adjusted with citric acid. To record light microscopy images, native and modified starches (0.8 g) were suspended in water (10 mL), and depending on the preparation, the starch slurry was incubated or heated at an appropriate temperature for 15 min with vigorous stirring. The temperature conditions, at which the slurry was treated, were determined based on the pasting characteristics of each individual preparation analysed with Brabender viscograph. The temperatures corresponding to the changes in the Brabender profile of the analysed sample, were used for the specimen preparation, and they were given in the captions of the figures presented in the work. A drop of the heated sample was transferred onto glass slides, smeared, and, following cooling, stained with Lugol’s solution (standard procedure). The preparations were examined with an OLYMPUS BX60 (Olympus, Japan) microscope.

Degree of substitution of food grade modified starches were determined according to JECFA recommendations [23]. For cationic starches the degree of substitution was establish through nitrogen content determination according to EN ISO 3188 standard. Samples obtained in the microwave process were first purified with hydrochloric acid. To this aim, starch samples were suspended in 5% solution of hydrochloric acid, filtered, and washed with water to remove chloride ions and air dried.

## 3. Results and Discussion

### 3.1. Starch Gelatinisation

Starch in its native granular form has very limited applicability, as it is insoluble in cold water. Therefore, the process of its dissolution (gelatinisation or pasting) is a subject of keen interest of technologists. Standard methods for the study of the pasting process involve using viscographs, such as Brabender viscograph or Rapid Visco Analyser. They provide useful information regarding changes in viscosity of starch dispersion in water during heating and cooling. Quantitatively, the process of gelatinisation is studied with differential scanning calorimetry. This technique provides information on the temperature-dependent enthalpy changes that accompany the decay of the crystalline phase in starch granules [24]. However, both of these analytical methods provide no information on the molecular mechanism of the gelatinisation process. To some extent, light microscopy makes it possible to study this phenomenon.

**Figure 1:**
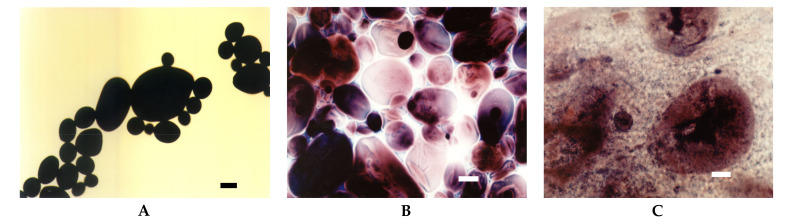
LM micrographs (20×) of native potato starch dispersions incubated at different temperatures for 15 min: (**A**) 25 °C, (**B**) 68 °C, (**C**) 90 °C, (Bar-20 µm). LM: light microscopy.

**Figure 2:**
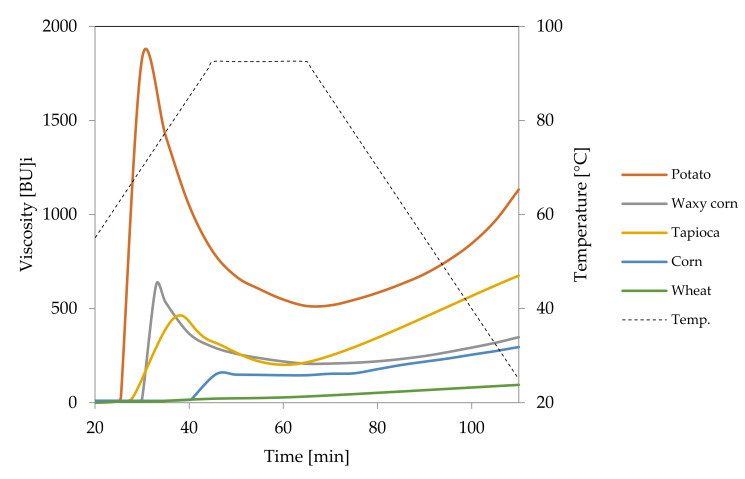
Pasting characteristics of native starches recorded for starch dispersions in concentration of 5%.

At room temperature potato starch granules remained unchanged and were visualised in the micrographs as black spots (Figure 1A). This corresponds to zero viscosity on the Brabender curve (Figure 2). At temperatures above 60 °C, the gelatinisation process started which manifested as an increase in viscosity (Figure 2) as a result of swelling of starch granules. In LM images, swollen granules are visible as red-blue-mazarine ovals (Figure 1B). Red colour corresponds to amylopectin-iodine complexes, blue–to amylose complexes, darker parts are non-gelatinised. The degree of transparency of these shapes depended on the degree of gelatinisation of different granules. Moreover, the hydrated amylose phase that start to leak out from the starch granules can be also visible forming the paste milieu (Figure 1B). The first stage of gelatinization of potato and tapioca starches was captured with SEM where amylose leakage was also noted [7]. The gelatinisation stage of potato starch granules seems to be more advanced upon their heating at 90 °C (Figure 1C). A substantial part of the released amylose appeared as droplets and together with solubilised, to some extent, amylopectin phase formed a bicontinous network.

**Figure 3:**
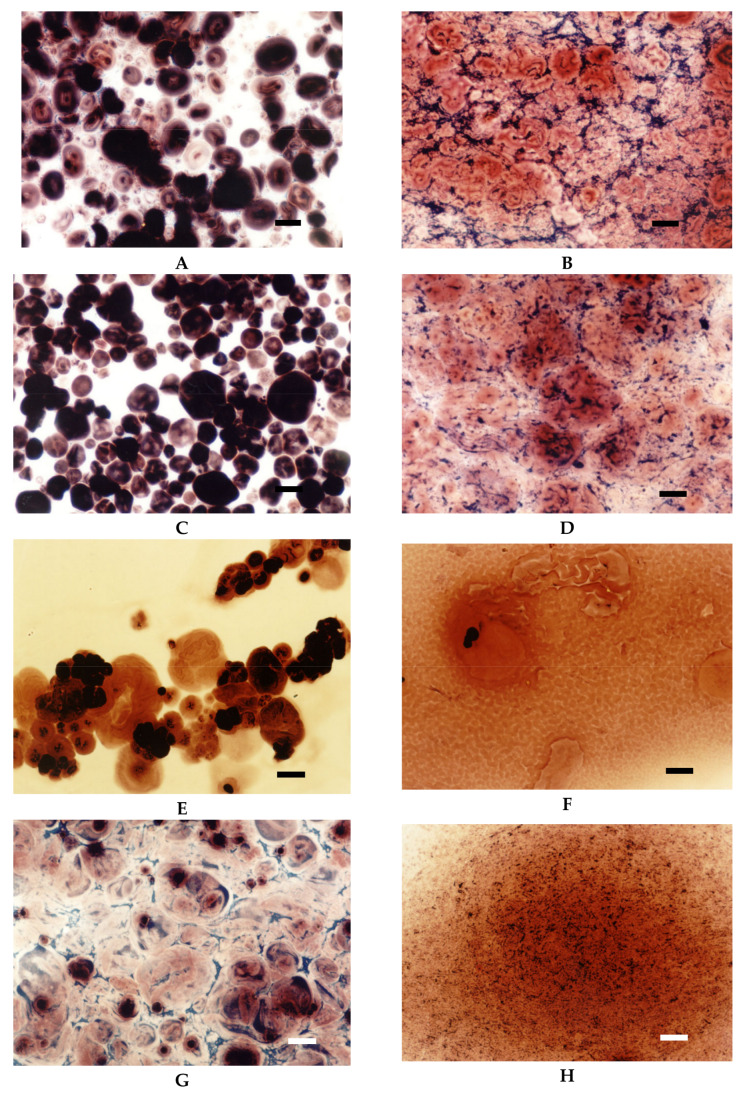
LM micrographs (20×) of native starch dispersions incubated at different temperatures for 15 min: (**A**) Wheat 75 °C, (**B**) Wheat 90 °C, (**C**) Corn 75 °C, (**D**) Corn 90 °C, (**E**) Waxy corn 68 °C, (**F**) Waxy corn 90 °C, (**G**) Tapioca 68 °C, (**H**) Tapioca 90 °C, (Bar-20 µm).

Light microscopy makes it also possible to visualise the differences between starches of various botanical origin. As presented in Figure 3, regular cereal starches revealed medium type of swelling characteristics (Figure 2), the first symptoms of gelatinisation–a slight swelling of the granules was observed at the temperature of 75 °C. In the case of waxy corn and tapioca starches, which manifest high type of swelling characteristics, advanced signs of gelatinisation were observed already at 68 °C. At 90 °C, significant differences could also be observed. Although none of the starch formed a homogeneous dispersion, in the case of tapioca and waxy corn starches, the pasting process was much more advanced than in regular cereal starches. Comparing Figure 3B–G, it can be stated that wheat starch at the temperature of 90 °C was at a similar stage of gelatinisation as tapioca starch at 68 °C. Incomplete gelatinisation of regular cereal starches corresponds to their low thickening capability and, as a consequence, is the reason for their uncompetitiveness as texture forming agents in food technology.

### 3.2. Starch Modification

#### 3.2.1. Pregelatinisation

**Figure 4:**
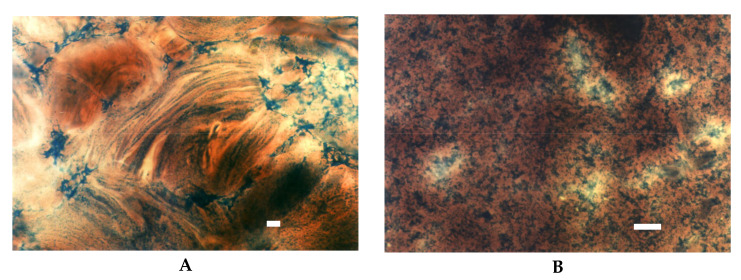
LM micrographs of pregelatinised starch dispersions incubated at a temperature of 25° for 15 min: (**A**) drum dried (40×), (**B**) extruded, (20×), (Bar-20 µm).

Many methods of starch modification have been developed in order to enhance the positive attributes and eliminate the shortcomings of the native starches [25]. The most common type of modification in industrial practice is the production of pregelatinised starch. It involves gelatinisation and drying stages and is typically performed using drum dryers. Nevertheless, it is also possible to use other types of apparatus, such as extruders. As a result of the demand from food industry, not only native but also chemically modified starches undergo this type of processing. Pregelatinisation results in complete disruption of starch granules. The product takes the form of white flakes which has been documented using scanning electron microscope [26]. Commonness of the technological use of pregelatinised starch stems from its ability to form paste without heating. It should be emphasised that the homogeneity of dispersions of pregelatinised starch (Figure 4) was similar to that of cooked starch (Figure 1C).

Significant differences were visible between drum dried and extruded starches. Both of these starches reveal lower viscosity when compared to their not processed counterpart. However, this difference is more pronounced in extruded starch [27]. LM pictures support this observation (Figure 4). As visualised in the image of drum dried starch (Figure 4A) the effect of shearing stress that starch paste was subjected resulted in partially parallel setting of high molecular starch molecules. In contrast, starch macromolecules degraded by extrusion did not succumb to the ordering action of shear forces (Figure 4B).

#### 3.2.2. Chemical Modification

The reactivity of hydroxyl groups present in the structure of starch macromolecules creates the possibility for various chemical reactions which results in a vast range of modified starches. Despite the enormous variety of modified starches offered on the market, the applied chemical reactions can be classified to three types: oxidation, esterification, and etherification. The use of multi-functional reagents (mainly bifunctional) enables cross-linking of starch macromolecules in the course of esterification or etherification. This creates almost limitless possibilities of precise tuning of the properties of modified starches to specific applications [25].

##### Oxidation

**Figure 5:**
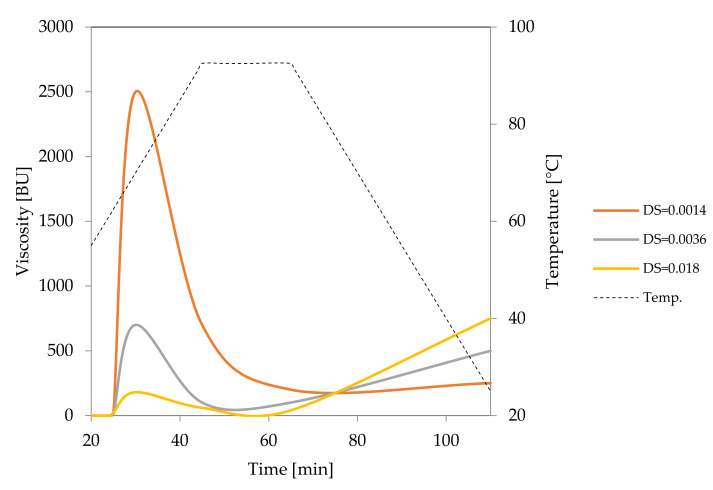
Pasting characteristics of oxidised starches recorded for starch dispersions in concentration of 8% (DS = 0.0014 and DS = 0.0036) or 20% (DS = 0.018).

Oxidation of starch can be performed with different agents, such as hydrogen peroxide, ozone or sodium periodate. Nonetheless, the process that employs sodium hypochlorite is of the greatest practical importance. Hypochlorite modified starches were initially used primarily as sizing agents [28]. Nevertheless, they are currently widely used as texture-forming ingredients in the food industry. According to Codex Alimentarius, the degree of substitution of hypochlorite modified starches for food application should not exceed 0.05 [29]. Actually, most modified starches offered on the market have a degree of substitution well below this limit, and some of them meet the requirements of bleached starches. The oxidation reaction is performed in suspension which enables accurate removal of unreacted substrates and by-products. SEM studies proved that it does not cause significant changes in the morphology of starch granules [28]. However, hypochlorite processing is accompanied by a significant reduction in viscosity. The decrease in viscosity is so deep that it was necessary to study the course of pasting at higher concentrations compared to native starches (Figure 5). With the increase of the degree of oxidation, an increase in the final viscosity of the pastes accompanied by a decrease in the peak viscosity was observed. This is a result of the increasing content of carboxyl groups which promote the disintegration of granules during heating. During cooling, however, these groups drive the association of starch macromolecules and gel formation. For this reason, oxidised starches reveal gelling properties that make them highly popular in food production.

**Figure 6:**
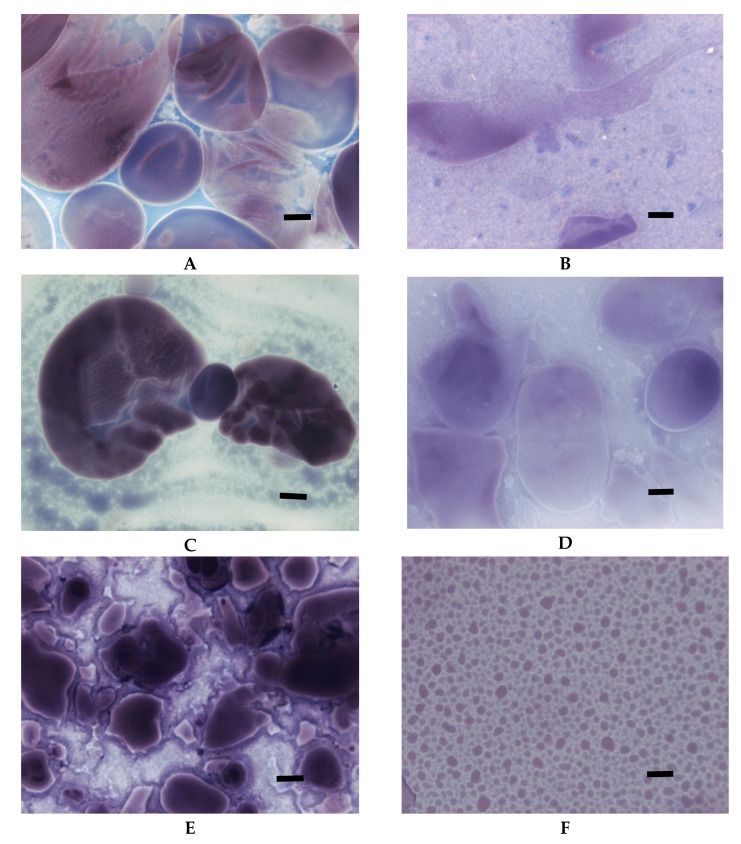
LM micrographs (20×) of oxidised starch of potato origin with different degrees of substitution incubated at initial and advanced stages of gelatinisation: (**A**) DS = 0.0014; 65 °C, (**B**) DS = 0.0014; 90 °C, (**C**) DS = 0.0036; 65 °C, (**D**) DS = 0.0036; 90 °C, (**E**) DS = 0.018; 65 °C, (**F**) DS = 0.018; 90 °C, (Bar-20 µm).

Changes in the starch pasting process and in the starch-water interactions that were related to the oxidation of starch were also reflected in the LM images (Figure 6). At the temperature of 65 °C, slightly oxidised (DS = 0.0014) starch (Figure 6A) revealed more advanced gelatinisation than native potato starch (Figure 1B). However, the basic pattern of this process did not change as swollen starch granules suspended in amylose solution could be observed. As oxidation increased, not only swelling but also disintegration of starch granules was observed at the temperature of 65 °C (Figure 6C,E). At the temperature of 90 °C, starches of degree of oxidation of 0.0014 and 0.0036 formed dispersions similar to those of native starch. Due to significantly reduced viscosity, the most oxidized starch formed untypical dispersion in which molecules of amylose and amylopectin could no longer be distinguished (Figure 6F).

However, the most important observation coming from the LM studies of oxidised starch is probably the one concerning colour upon iodine staining. The colour was significantly altered in comparison to micrographs of native starch. This observation is important because of the popularity of iodine colorimetry for determination of amylose content [30]. Substituents built into the structure of macromolecules significantly change the ability to form the complex with iodide and can cause false results of analyses.

##### Esterification-Acetylation

**Figure 7:**
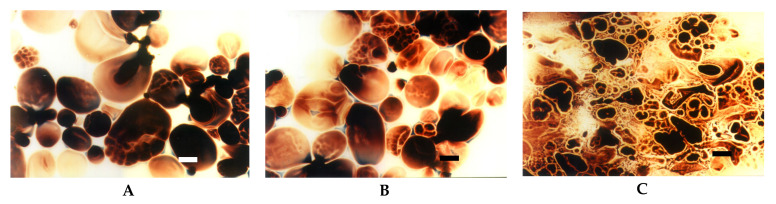
LM micrographs (20x) of acetylated starch of potato origin with different degrees of substitution incubated at the temperature of 60 °C: (**A**) DS = 0.008, (**B**) DS = 0.018, (**C**) DS = 0.123 (Bar-20 µm).

Acetylation of starch, like oxidation, has been known for over 150 years and still is of industrial importance. This reaction is applied either to obtain exclusively acetylated starches or dually modified acetylated crosslinked derivatives. Acetylation performed in anhydrous media makes it possible to achieve a very high degree of substitution, up to 2.9. For this reason, starches modified by this method have significantly changed morphology, decreased crystallinity, and are soluble in non-polar solvents [31]. For application in food, however, the process is accomplished in water suspension to obtain a degree of substitution not higher than 0.1 [29]. Such starches do not reveal significant changes in morphology [32]. Changes in the rheological properties include decreased pasting temperature and increased shear resistance with almost no change in viscosity [33,34]. LM studies proved that the incorporation of acetyl groups into the starch macromolecules resulted in enormous changes in starch-water interactions (Figure 7).

Even by the presence of less than one acetyl group per hundred anhydroglucose units, no more blue colour that corresponds to amylose-iodine complex could be observed (Figure 7A). This observation points even more strongly to the issue of the possible lack of reliability of the iodine colorimetry method [30] in the determination of amylose content in modified starches. The presence of acetyl groups changes iodine complexation and may cause incorrect results of analyses even more strongly than the presence of carboxyl groups. With increasing degree of substitution, additional disintegration of starch granules was observed (Figure 7B,C). LM observations suggest that the presence of relatively hydrophobic acetyl groups leaded to significant changes in the conformation of starch macromolecules in a water colloidal system. This hypothesis has been supported by the studies employing gel permeation chromatography with triple detection. The increase of the degree of substitution with acetyl groups causes a slight decrease in molecular mass accompanied by a significant change of the conformation of starch molecules towards decreased sphericity [35]. Moreover, the dynamics of water molecules in acetylated starch pastes, studied with low field nuclear magnetic resonance, were found to decrease with an increase of the degree of substitution [36].

##### Esterification–Crosslinking

**Figure 8:**
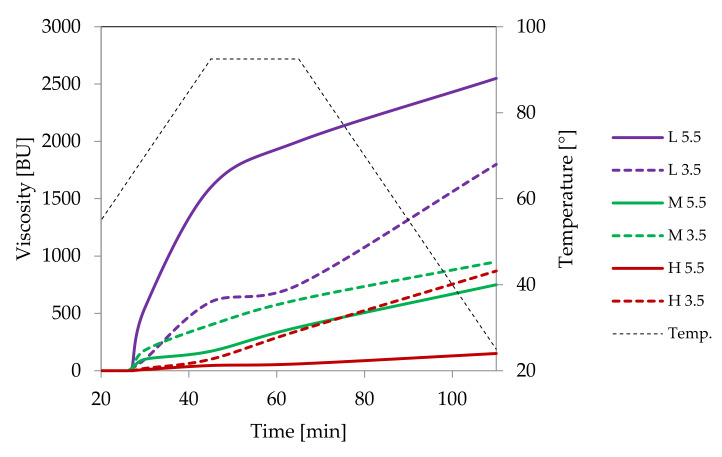
Pasting characteristics of samples of distarch phosphate of potato origin with different degrees of crosslinking (L–low, M–medium, H-high) recorded for dispersions in a concentration of 4.5% at pH = 5.5 or pH = 3.5.

**Figure 9:**
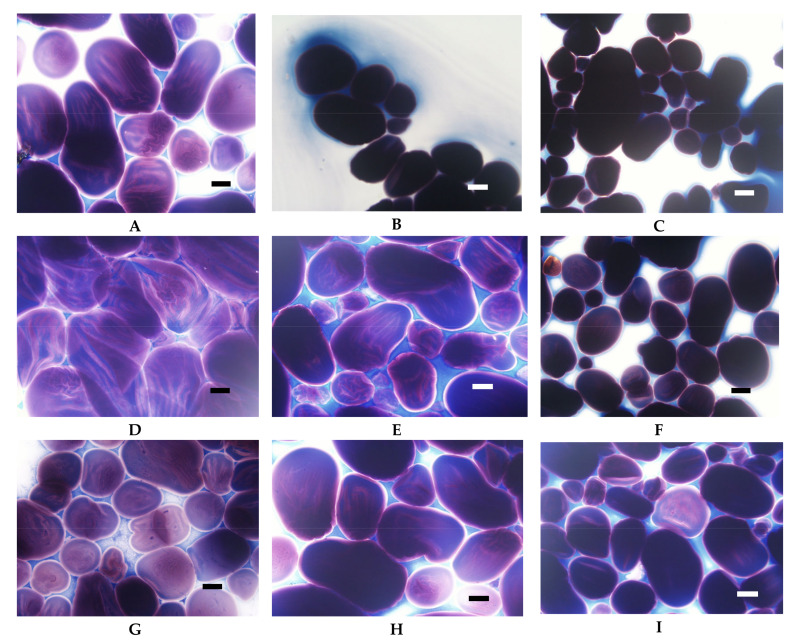
LM micrographs (20×) of distarch phosphates of potato origin with different degrees of crosslinking (L–low, M–medium, H-high) incubated in different conditions: (**A**) L, 90 °C, pH = 5.5; (**B**) M, 90 °C, pH = 5.5, (**C**) H, 90 °C pH = 5.5; (**D**) L, 123 °C, pH = 5.5; (**E**) M, 123 °C, pH = 5.5, (**F**) H, 123 °C, pH = 5.5; (**G**) L, 90 °C, pH = 3.5; (**H**) M, 90 °C, pH = 3.5, (**I**) H, 90 °C pH = 3.5 (Bar-20 µm).

**Figure 10:**
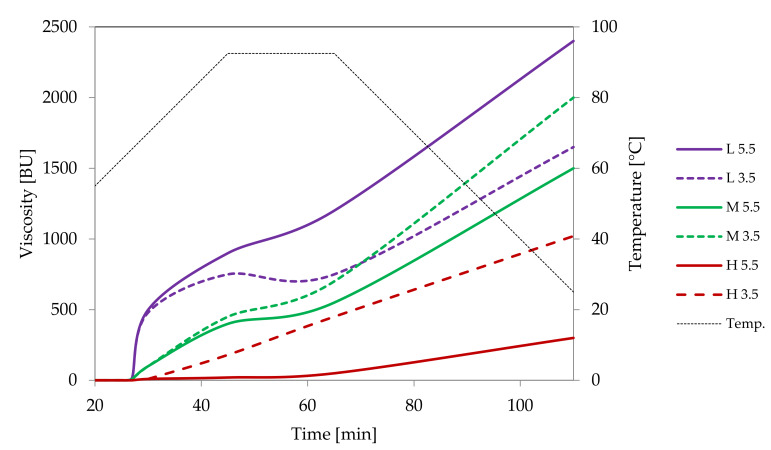
Pasting characteristics of acetylated distarch phosphate of potato origin with different degrees of crosslinking (L–low, M–medium, H-high), recorded for dispersions of the concentration of 4.5% at different pH.

**Figure 11:**
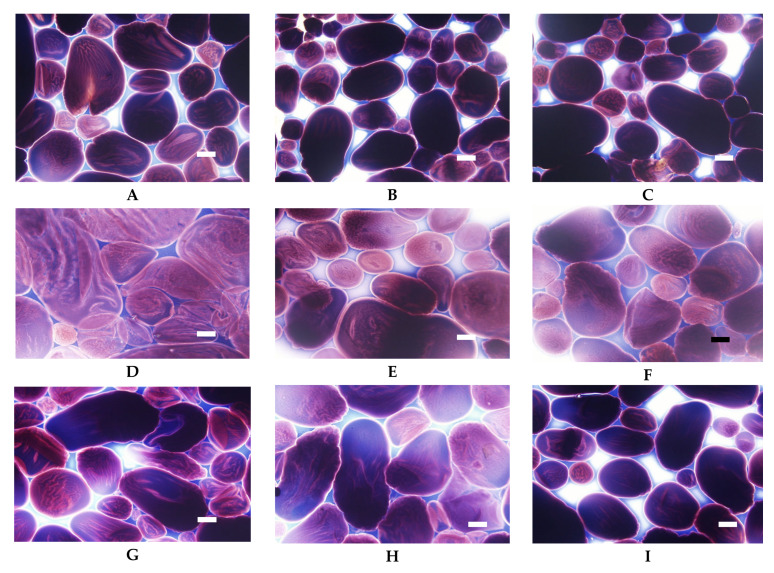
LM micrographs (20×) of acetylated distarch phosphates of potato origin with different degrees of crosslinking (L–low, M–medium, H-high) incubated in different conditions: (**A**) L, 90 °C, pH = 5.5; (**B**) M, 90 °C, pH = 5.5, (**C**) H, 90 °C pH = 5.5; (**D**) L, 123 °C, pH = 5.5; (**E**) M, 123 °C, pH = 5.5, (**F**) H, 123 °C, pH = 5.5; (**G**) L, 90 °C, pH = 3.5; (**H**) M, 90 °C, pH = 3.5, (**I**) H, 90 °C pH = 3.5 (Bar-20 µm).

Crosslinked starches are readily used in food production. A very slight change in the structure of starch macromolecules causes huge changes in the properties of this biopolymer. The degrees of substitution of food grade crosslinked starches are about an order of magnitude lower than those of monostarch derivatives [29]. Crosslinking causes an increase in the molar mass of starch which initially results in an increase in viscosity. However, further increasing of the degree of crosslinking results in the formation of molecules with limited solubility. As a consequence, high paste viscosity gradually decreases until, finally, starch becomes completely insoluble. Crosslinking of starch for food applications with sodium trimetaphosphate does not change the crystallinity of starch and the morphological changes are surface related mainly because of the action of alkali present in the suspension [25,32]. That type of processing is intended to make starch pastes more resistant to extended cooking times, increased acidity or shear stress [32]. By controlling the degree of substitution of crosslinked starches, precise tuning of their properties for specific applications is possible (Figure 8, Figure 9, Figure 10 and Figure 11).

Low cross-linked distarch phosphate, the most popular variant of E1412 in food technology, revealed higher viscosity than native potato starch in pH = 5.5. Microscopically, it was manifested by the formation of a paste at the temperature of 90 °C (Figure 9A) that was visually similar to the paste of native starch incubated at 68 °C and which features are characteristic of the initial phase of gelatinisation. A more advanced process of gelatinisation could be observed after sterilisation (Figure 9D). This indicates the special suitability of this type of starch for thickening of food processed by sterilisation. In acidic conditions starch granules appeared to be less swollen than at a pH of 5.5. However, the extent of amylose leakage from the granules was greater (Figure 9G). This corresponded to decreased viscosity of the paste (Figure 8). Nonetheless, this decrease was smaller than the one observed in native starches [21]. Following incubation at the temperature of 90 °C and the pH = 5.5, medium and highly crosslinked starches remained almost ungelatinised (Figure 9B,C). This corresponded to a significant decrease in viscosity (Figure 8). Medium crosslinked distarch phosphate was partially gelatinised in sterilisation (Figure 9E) and in acidic conditions at 90 °C (Figure 9H). Highly cross-linked starch underwent this process only at a pH of 3.5. It is noteworthy that the viscosity of medium and highly crosslinked distarch phosphates was higher in acidic than in neutral environment (Figure 8). These observations are direct indications of the technological suitability of STMP crosslinked starch.

Acetylated distarch phosphate samples revealed gelatinisation patterns similar to their non-acetylated counterparts (Figure 10 and Figure 11). When compared to the non-acetylated variant, a smaller decrease in the viscosity of low cross-linked samples pasted at pH = 3.5 is noticeable (Figure 10). This points to an even better suitability of dually modified starch phosphate for the stabilisation of acidic food products in comparison to distarch phosphate. The presence of acetyl groups slightly facilitated the gelatinisation process (Figure 11). Nevertheless, highly cross-linked acetylated distarch phosphate showed a low degree of pasting under all conditions used.

##### Etherification

**Figure 12:**
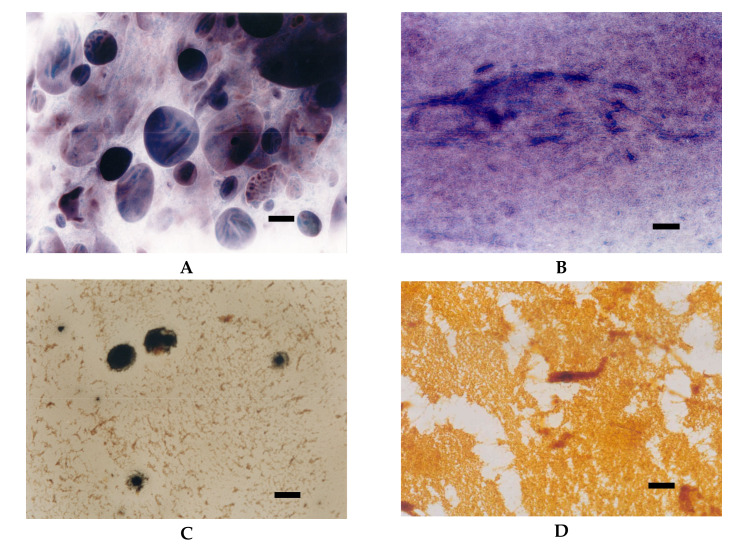
LM micrographs (20×) of potato cationic starches with different degrees of substitution incubated at different temperatures: (**A**) DS = 0.04, 60 °C; (**B**) DS = 0.04, 90°C; (**C**) DS = 0.13, 25 °C; (**D**) DS = 0.27, 25 °C (Bar-20µm).

Starch modified by the etherification reaction are primarily hydroxypropylstarch used both in food production and other industries as well as cationic starches designed for paper sizing. Hydroxypropylation results in a significant decrease in pasting temperature, time to attain peak viscosity accompanied by increased solubility, and paste clarity. Moreover, hydroxypropylated starches reveal excellent freeze-thaw stability as well as reduced retrogradation tendency which is important in food applications. SEM studies proved that hydroxypropylation results in deep changes in the morphology of starch granules, including the main alteration – formation of deep grooves in the central core region. The extent of these changes depends on the amount of the hydroxypropyl groups incorporated into starch macromolecules [32,37,38]. The effect of the degree of substitution on the morphology of starch granules is observed also in the case of cationic starches. A distinguishing feature of cationic starches is the presence of positively charged quaternary ammonium groups in the structure of starch macromolecules. Due to strong electrostatic interactions between cationic starch and cellulose fibres, it is an excellent preparation for improving the quality of paper, mainly its mechanical properties [22,39]. Cationic starches with a degree of substitution up to DS = 0.04 can be manufactured in water suspension. That type of starches does not show changes in the morphology of granules or crystal structure, but its pasting temperature is lower and the time to attain peak viscosity is shorter. Moreover, starches that reveal medium type of pasting characteristics in their native form (corn, wheat, pea), change it into high type as a result of cationisation. The presence of cationic groups also causes conformational changes in starch macromolecules. Electrostatic repulsion increases the volume of a starch coil in solution, which is manifested by shorter retention times during size exclusion chromatography “SEC” analysis in comparison to native starch [22]. The LM micrographs of cationic starch with DS = 0.04 were similar to those of oxidised starch (Figure 12A,B). Compared to native starch, facilitation of the pasting process as well as different mode of iodine complexation were observed.

To obtain products with higher degrees of substitution, dry or semi dry reaction conditions are required. Starches subjected to such conditions not only change their morphology but also lose their crystal structure [22,39]. The presence of a large number of cationic groups improves dissolution of starch (Figure 12C,D). By substitution of every fourth anhydroglucose unit, starch became completely soluble in room temperature (Figure 12D). Moreover, conformational changes caused by the presence of cationic groups changed the mode of iodine complexation and, in consequence, the colour of starch pastes observed in the LM images.

## 4. Conclusions

Despite the great progress in microscopy, visual light microscopy remains a potentially useful technique for studying the structure and functionality of starch. While advanced microscopic techniques, such as AFM, CLSM or SEM, provide more detailed information regarding the morphology of the starch granule, LM can be successfully used to demonstrate the structural changes of starch in dispersed systems. With the use of LM, amylose can be easily distinguished from amylopectin by iodine staining. Moreover, the extent of molecular dispersion of starch in pastes as well as the changes in the polymer-solvent interaction caused by the substitution of hydroxyl groups with polar or non-polar moieties can also be successfully studied under LM. These features make the technique particularly useful for investigating the changes in the supramolecular structure of starch in the matrix of food products as they allow to obtain essential and detailed information.
